# Intraosseous synovial sarcoma of the distal ulna: a case report and review of the literature

**DOI:** 10.1186/s12885-019-5325-x

**Published:** 2019-02-01

**Authors:** Taketsugu Fujibuchi, Joji Miyawaki, Teruki Kidani, Hiroshi Imai, Hiroshi Kiyomatsu, Riko Kitazawa, Hiromasa Miura

**Affiliations:** 1 Department of Bone and Joint Surgery, Ehime University Graduate School of Medicine, Shitsukawa, Toon, Ehime 791-0295 Japan; 2 Department of Molecular Pathology, Ehime University Graduate School of Medicine, Shitsukawa, Toon, Ehime 791-0295 Japan

**Keywords:** Synovial sarcoma, Bone tumor, SYT-SSX fusion gene

## Abstract

**Background:**

Synovial sarcoma is a relatively rare type of soft tissue sarcoma. The commonly observed symptom is a deep-seated palpable mass accompanied by pain or tenderness. Thus, it is considered a soft tissue sarcoma and rarely occurs primarily in bone. However, only few studies have been reported on intraosseous synovial sarcoma, and reports on cases with cytogenetic or molecular confirmation are even rarer. We report a case of intraosseous synovial sarcoma of the distal ulna that has been confirmed using histopathological examination and molecular analysis.

**Case presentation:**

A 77-year-old female was referred to our hospital with a 1-month history of right wrist pain after housework. Clinical and imaging findings suggested a benign bone tumor that was enhanced by Gd-DTPA. It was thought that the tumor was possibly an enchondroma. Initially, we planned to evaluate the benignancy of the tumor with intraoperative frozen section, followed by curettage and bone graft at one stage However, when considering carefully, characteristics of the tumor did not perfectly match those of any diagnostic categories including enchondroma. Therefore, an incisional biopsy was performed and revealed that the tumor was synovial sarcoma. Following an elaborate plan, the patient underwent a wide resection of the tumor at the distal part of the right ulna. Reverse transcription-polymerase chain reaction (RT-PCR) from the resected specimen and sequencing of RT-PCR products demonstrated a chimeric SYT-SSX1 transcript, confirming the diagnosis of synovial sarcoma.

**Conclusions:**

Synovial sarcoma is seldom considered in differential diagnosis of bone tumors because it is difficult to line up such an unusual diagnosis as a differential diagnosis. When the lesion does not perfectly fit into any diagnostic category, when the initial image diagnosis appears unconvincing, biopsy and pathology are indicated, recalling Jaffe’s triangle. According to these diagnostic processes, the patient successfully completed the treatment for this rare intraosseous synovial sarcoma, following a careful plan based on the preoperative diagnosis.

## Background

Bone and soft tissue tumors do not always show typical clinical presentations or imaging findings. Although many bone tumors can be diagnosed using clinical and imaging findings, clinicians at times encounter a case where these findings do not fit into any diagnostic category. In such cases, it becomes crucial to comprehensively consider clinical, radiological, and pathological findings. Skipping this process and making an uncertain diagnosis increases the risk of unsatisfactory patient outcomes.

Synovial sarcoma is a relatively rare type of soft tissue sarcoma. It is prevalent in adolescents and young adults, occurring predominantly in the extremities, especially in the lower extremities. The commonly observed symptom is a deep-seated palpable mass accompanied by pain or tenderness. Radiographic examinations often reveal calcification of the tumor. Histologically, synovial sarcoma is classified as biphasic or monophasic type. It displays a variable degree of epithelial differentiation, including glandular structures, and it is characterized by a specific chromosomal translocation t (X; 18) (p11; q11) that leads to the SYT-SSX fusion gene formation [1, 2].

Synovial sarcoma usually occurs primarily in the soft tissue and rarely in bone. However, only few studies have been reported on intraosseous synovial sarcoma [3–14], and reports on cases with cytogenetic or molecular confirmation are even rarer [3, 4, 9–11, 14]. Here, we report a case of intraosseous synovial sarcoma of the distal ulna. Although on imaging the tumor resembled a benign bone tumor, when considering carefully, the tumor did not perfectly match those of any diagnostic categories. Therefore, preoperative histopathological examination following incisional biopsy revealed the tumor to be synovial sarcoma. The patient underwent a planned surgery, and molecular analysis of the SYT-SSX1 fusion gene confirmed the diagnosis of synovial sarcoma.

## Case presentation

**Fig. 1:**
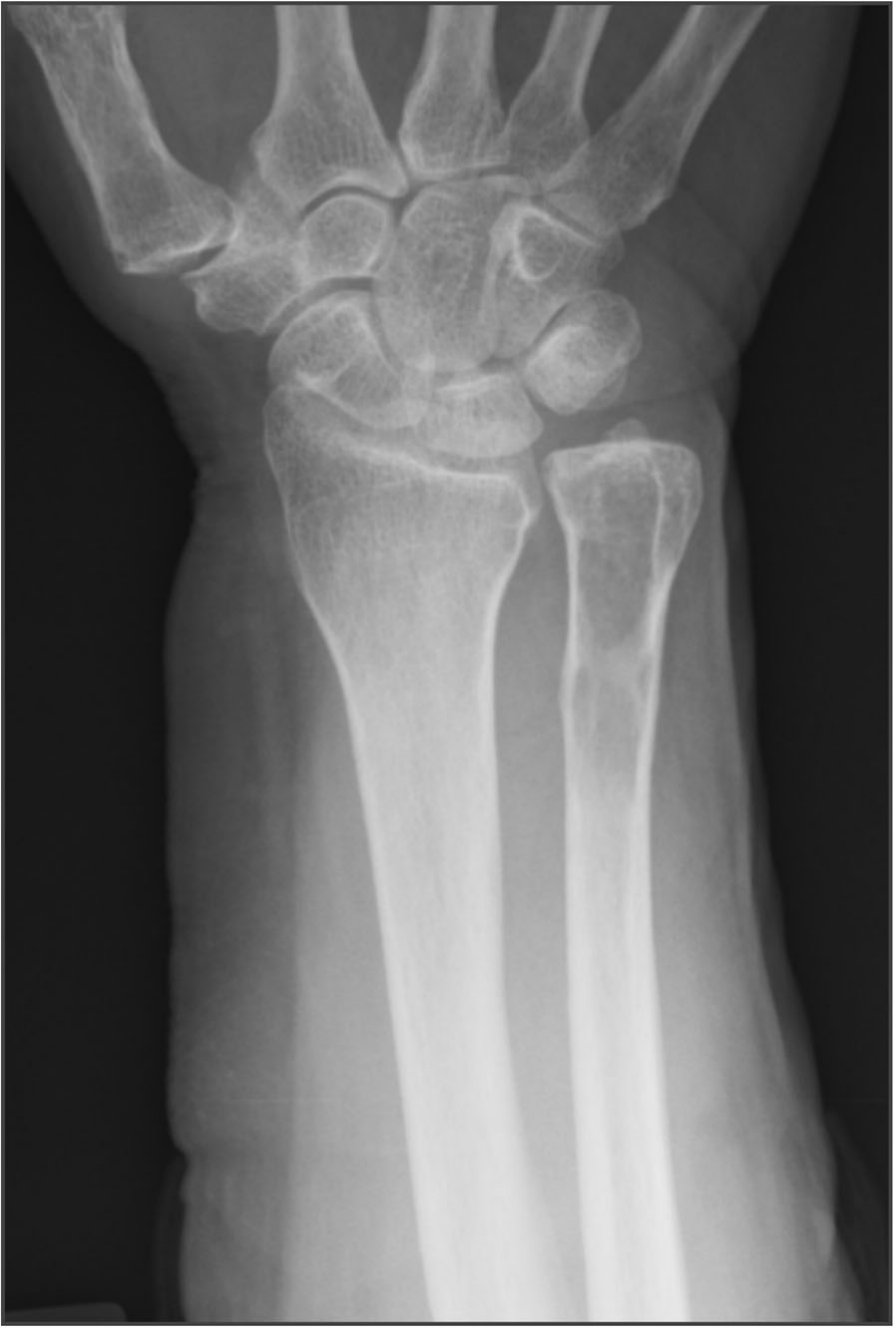
An anteroposterior plain radiograph of the right wrist. There is a comparatively well outlined osteolytic lesion at the distal end of the ulna

**Fig. 2:**
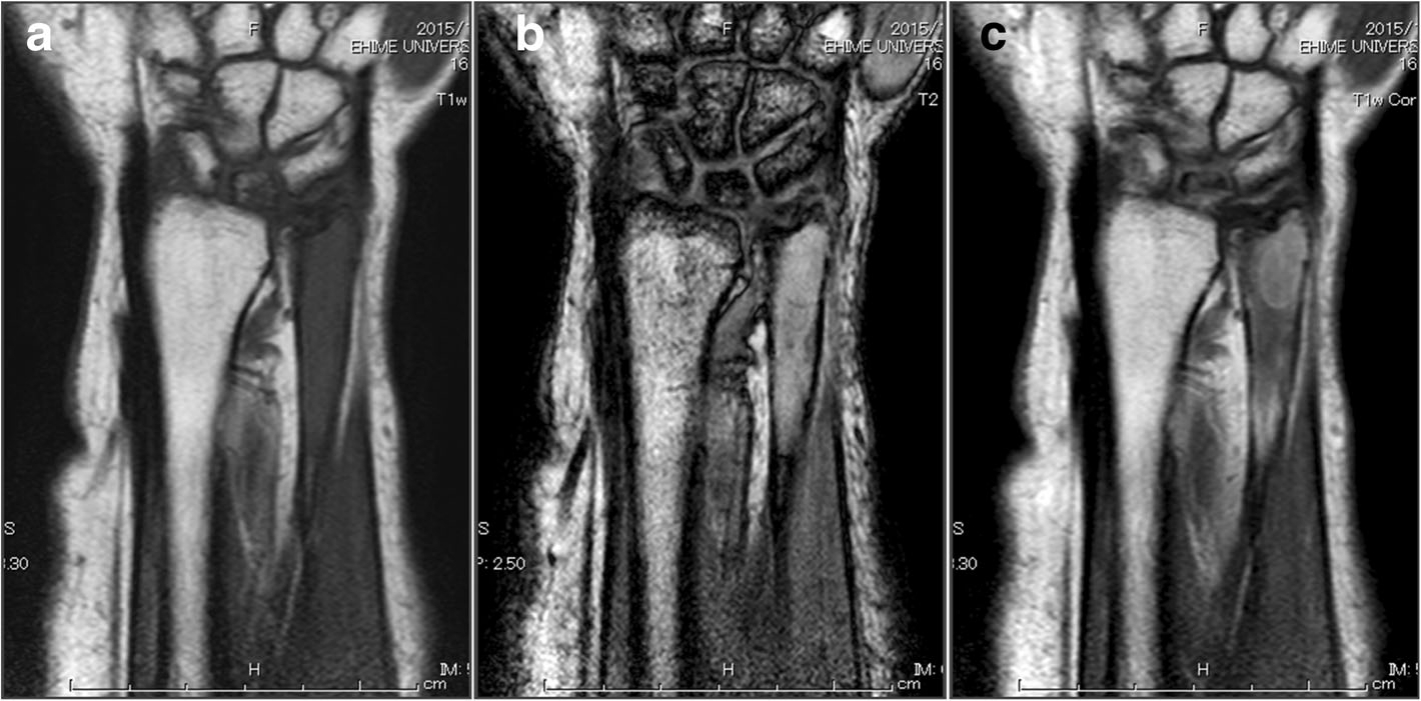
MRI of the lesion of the distal ulna. **a** The mass showed iso-intensity on T1-WI, **b** almost homogenous high intensity on T2-WI, a low intensity line in the proximal end of the lesion suggesting sclerotic rim, and **c**. was enhanced heterogeneously by Gd.-DTPA. The lesion stayed inside the distal ulna bone; there were no extraosseous masses

**Fig. 3:**
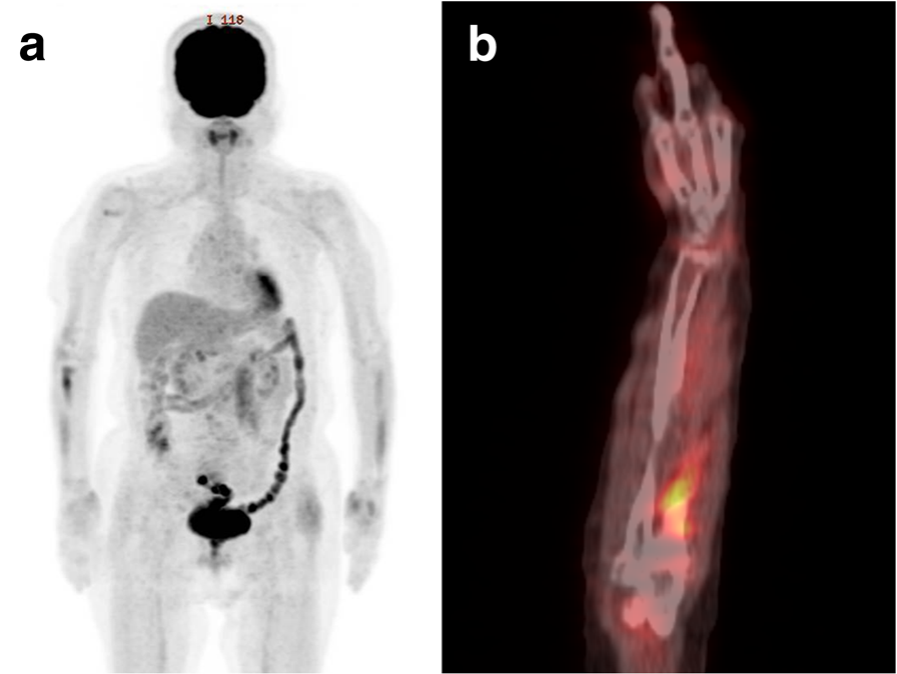
PET-CT imaging. **a** Whole body scan did not show any distant metastasis nor abnormal FDG uptake in the distal ulna. **b** Scan of forearm showed no abnormal FDG uptake in the lesion

A 77-year-old female was referred to our hospital with a 1-month history of right wrist pain after housework. She had a medical history of hypertension, dyslipidemia, and no particular notable family history. During physical examination, she reported a slight pain and tenderness in the ulnar side of her right wrist. The swelling or mass were not palpable. Range of motion of the right wrist was slightly disturbed. Plain radiography revealed a comparatively well-outlined osteolytic lesion in the distal end of the ulna (Fig. 1). Magnetic resonance imaging (MRI) also demonstrated a bone tumor in the distal end of the ulna. The mass showed iso-intensity on T1-weighted images (T1-WI), high intensity on T2-weighted images (T2-WI), and was heterogeneously enhanced by gadolinium-diethylenetriaminepentaacetic acid (Gd.-DTPA) (Fig. 2). No extraosseous masses were observed. Positron emission tomography-computed tomography (PET-CT) showed no abnormal fluorodeoxyglucose (FDG) uptake in the lesion (Fig. 3). No distant lesions, including lung lesions were noted. Clinical and imaging findings suggested a benign bone tumor that was enhanced by Gd.-DTPA. It was thought that the tumor was possibly an enchondroma. Initially, we planned to evaluate the benignancy of the tumor with intraoperative frozen section, followed by curettage and bone graft at one stage. However, when considering carefully, characteristics of the tumor did not perfectly match those of any diagnostic categories including enchondroma. In the case of enchondroma, it usually shows no significant enhancement or only marginal enhancement by Gd.-DTPA, however, the whole lesion was heterogeneously enhanced in this case. Therefore, an incisional biopsy was performed.

**Fig. 4:**
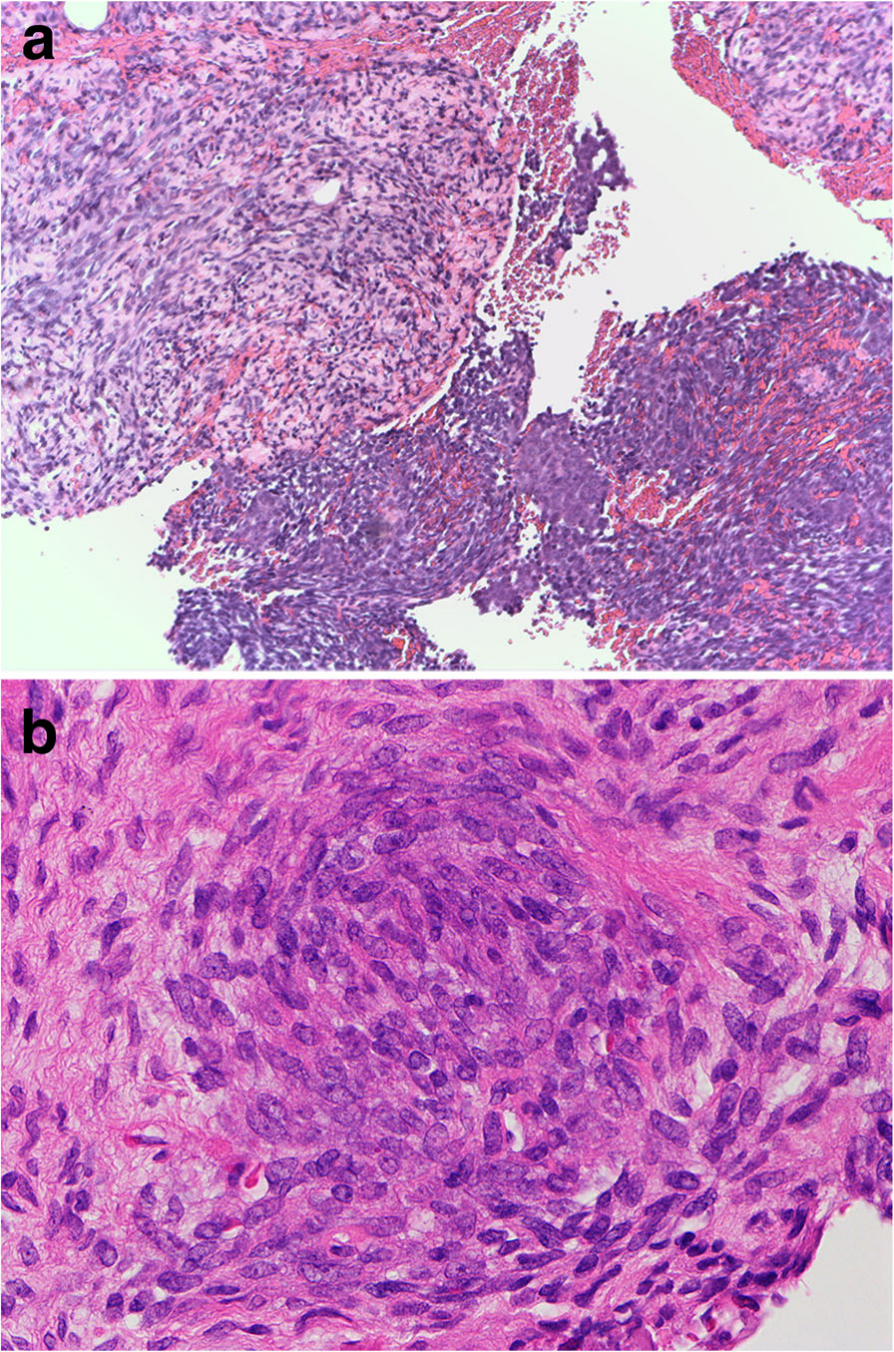
Histopathological appearance of the tumor. The specimen was composed of fascicles of spindle cells in a palisading pattern. Most of the lesion showed less aggressive pattern (the left side of **a**.); however, there was an area that showed partial high cellularity and nuclear atypia (the right side of **a**, **b**). Histologic features and immunohistochemistry results suggested a synovial sarcoma. Original magnification; **a**. × 100, b. × 400

Incisional biopsy revealed that the tumor comprised atypical spindle cells with hyper-cellularity (Fig. 4). The tumor cells were partially positive for epithelial membrane antigen and positive for B-cell leukemia/lymphoma 2 (Bcl-2) protein. Thus, synovial sarcoma was diagnosed based on histologic features and immunohistochemical results, though fluorescence in situ hybridization (FISH) examination filed to detect a rearrangement of SYT from the biopsy specimen.

**Fig. 5:**
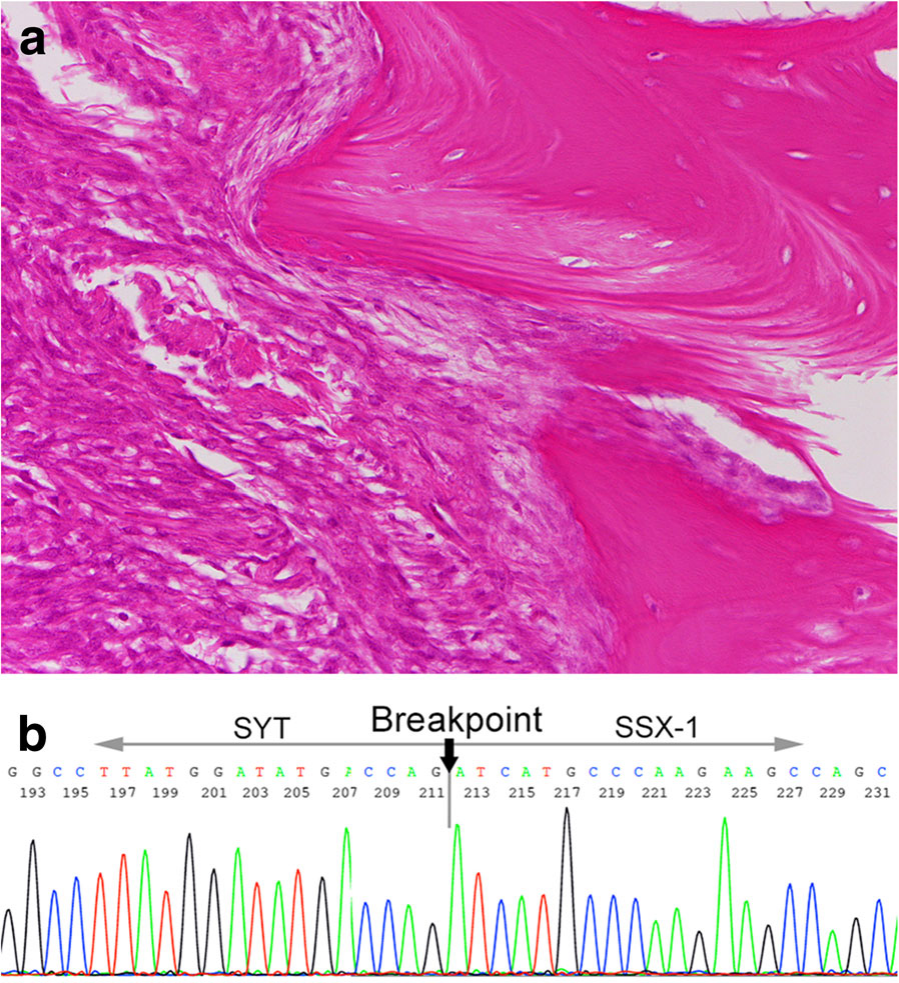
Postoperative analysis of resected tumor. **a** Histopathologically, the tumor consisted of spindle cells in a palisading pattern, and occupied the distal end of the ulna. Original magnification; × 200, **b** Molecular analysis definitely demonstrated the diagnosis of synovial sarcoma by confirming the SYT-SSX fusion gene. The DNA sequence data was identical to the alignment of SYT-SSX fusion gene, including the SSX1 breakpoint

Following an elaborate plan, the patient underwent a wide resection of the tumor at the distal part of the right ulna with biopsy tract. Any reconstructive soft tissue procedure was not performed. Histopathologically, the tumor occupied the distal end of the ulna, and demonstrated similar characteristics as the specimen obtained from the biopsy (Fig. 5a). Reverse transcription-polymerase chain reaction (RT-PCR) from the resected specimen and sequencing of RT-PCR products demonstrated a chimeric SYT-SSX1 transcript (Fig. 5b), confirming the diagnosis of synovial sarcoma.

At the 2-year follow-up, the patient is progressing favorably with 25 points on the Disability and Symptom section of Disabilities of the Arm, Shoulder, and Hand outcome measure and 93 points on the Toronto Extremity Salvage Score, with no evidence of local recurrence, distant metastasis or lung metastasis.

## Discussion and conclusions

Synovial sarcoma is a relatively rare malignant soft tissue tumor that accounts for 5–10% of all soft tissue sarcomas. Approximately 70% of synovial sarcomas occur in the deep soft tissues of the lower and upper extremities, often at a juxta-articular location; thus, it is considered a soft tissue sarcoma [1, 2]. The male and female external and internal sex organs, kidneys, adrenal glands, retroperitoneum, visceral structures, mediastinum, central nervous system, and peripheral nerves are unusual sites of involvement; bone is an even more unusual site. In cases where synovial sarcoma occurs in these rare sites, a definitive diagnosis is difficult and usually requires confirmation using cytogenetic or molecular analyses [2]. There have been only few reports regarding intraosseous synovial sarcoma with cytogenetic or molecular confirmation [3, 4, 9–11, 14]. In total, nine cases of intraosseous synovial sarcoma, with or without cytogenetic or molecular analyses, have been reported (Table 1) [3, 4, 8–14]. A case series and some case reports of primary intraosseous synovial sarcoma of the jaw [5, 6], and a case series of sacral synovial sarcoma were reported [7]; however, details of their imaging and histologic findings were unclear. To the best of our knowledge, our case is only the seventh reported case of intraosseous synovial sarcoma that has been confirmed using molecular analysis.

**Table 1:** Summary of previously reported cases of intraosseous synovial sarcoma with cytogenetic/molecular confirmation

Reference (year)	Site	Age (years)	Gender	Image diagnosis	Cytogenetic study	X-p	MRI T1WI	MRI T2WI	MRI T1WI Gd.	Extraosseous mass	PET-CT
Cohen et al. (1997) [3]	Proximal tibia	22	M	N/A	Spectral karyotyping, FISH	Lytic lesion	N/A	N/A	N/A	(+) A small soft-tissue element	N/A
Hiraga et al. (1999) [4]	Distal radius	67	M	N/A	RT-PCR	Radiolucent lesion Indistinct margin	Low-intensity	N/A	N/A	(+) Expansion of the tumor to the sorrounding soft tissue	N/A
Nakajo et al. (2005) [8]	Sterunum	86	M	N/A (Malignant bone tumor)	N/A	N/A	Heterogenous iso-intensity	High-intensity	Heterogenous enhancement	(++) Multiocula and lobulated	N/A
O’Donnell et al. (2006) [9]	Proximal ulna	37	M	Ewing sarcoma	RT-PCR	Ill-defined lucency > Aggressive bone destraction	Iso-intensity with High-intensity area suggesting hemorrhage	High-intensity	N/A	(++) Large lobulated mass extending circumferentially around bone	N/A
Jung et al. (2007) [10]	Distal tibia	21	F	Chondrosarcoma, Chondroblastoma	RT-PCR	Mixed-density and irregular lesion	Heterogenous iso-intensity	Heterogenous iso- to low-intensity	Heterogenous enhancement	(++)	N/A
Beck et al. (2011) [11]	Proximal tibia	53	M	N/A	FISH	Round lucency with well-defined and less well-defined margin	N/A	N/A	N/A	(++) Tumor breaking through the cortex and extending into adjacent soft tissue.	FDG accumulation (+) (Recurrent tumor)
Zulkarnaen et al. (2012) [12]	Proximal femur	57	M	N/A	N/A	N/A	Low-intensity	N/A	N/A	(−)	N/A
Kim et al. (2013) [13]	Cervical spine	17	M	Langerhans cell histiocytosis, Osteosarcoma, Chondrosarcoma	N/A	Expansile oteolytic lesion	Low-intensity	High-intensity	Enhancement	(+)	N/A
Cao et al. (2014) [14]	Thoracic spine	26	M	Eosinophilic granuloma	FISH	Hypointense bony erosion	Low-intensity	Slightly high-intensity	N/A	(+−) Tumor entering spinal canal	N/A
Fujibuchi et al. (Current study)	Distal ulna	77	F	Enchondroma	RT-PCR	Comparatively well outlined osteolytic lesion	Iso-intensity	High-intensity	Heterogenous enhancement	(−)	FDG accumulation (−)

*N/A* not available

In the past nine reports, the patients’ age varied from 17 to 67 years. The tumor locations also varied and included the tibia, radius, ulna, femur, sternum or spine [3, 4, 8–14]. The reports concerned eight men and one woman, indicating that men were more prone to suffer from intraosseous synovial sarcoma. The lesions appeared osteolytic on plain radiography [3, 4, 9–11, 13, 14], low- or iso-intensity on T1-WI MRI, of variable intensity on T2-WI MRI [4, 8–10, 12–14], and were heterogeneously enhanced using Gd.-DTPA [8, 10, 13]. Extraosseous masses were also observed [3, 4, 8–11, 13, 14]. PET-CT findings have not been reported for primary lesions, although, these findings were reported in recurrent cases and demonstrated abnormal FDG accumulation [11] (Table 1). The imaging findings of our case were inconsistent with those previous reports, as the lesion showed a comparatively well-outlined rim on plain radiography, was without extraosseous mass, and did not accumulate FDG on PET-CT. In addition, the patient was slightly older than the age of the past report. What differential diagnosis would follow from such imaging findings? Of the reported cases, half do not describe an image-based diagnosis, the other half report diagnosis of the lesions as Ewing sarcoma, chondrosarcoma, osteosarcoma, chondroblastoma, or eosinophilic granuloma using clinical and imaging findings [9, 10, 13, 14]. In our case, the lesion looks benign based on plain radiographs and FDG-PET. The lesion was diagnosed as enchondroma reluctantly, though, there was some discrepancy, for instance, enhancement pattern on MRI. And this initial diagnosis ended up being inaccurate.

There are some reports about the diagnostic accuracy of MRI. Kransdorf et al. reported that MRI revealed sufficient characteristics to allow specific diagnosis in 27 (24%) of 112 cases, and it correctly suggested a malignancy in 11 (46%) of 27 pathologically-confirmed malignancies [15]. Jonathan et al. described the diagnostic efficacy and MRI value for distinguishing benign and malignant lesions with a sensitivity, specificity, positive predictive value, and negative predictive value of 78, 89, 65, and 94% for malignant tumors [16]. However, these are old reports and MRI methods have considerably evolved since their publication. Additionally, there are seldom any new reports regarding accuracy of image diagnosis. Although, the study did not aim to reveal the accuracy of image diagnosis, it referred to some articles evaluating the significance of subspecialty second-opinion consultation or interdisciplinary tumor center contributions to musculoskeletal image diagnosis. An article reported that subspecialty second-opinion consultations made an accurate diagnosis before pathologic confirmation in 334 (82%) of 407 cases of musculoskeletal lesions [17]. Another article reported that the descriptive diagnosis matched the histologically definitive diagnosis in 44 (76%) of 58 cases of benign bone tumors, and descriptive diagnosis corresponded to histology in 26 (51%) of 51 tumor-like lesions at an interdisciplinary tumor center [18]. However, image diagnosis has variable accuracy; approximately 20–30% of cases could not be diagnosed only using physical and imaging findings.

Synovial sarcoma is seldom considered in differential diagnosis of bone tumors because it is difficult to line up such an unusual diagnosis as a differential diagnosis. There are many cases of bone tumors where the patient’s age, medical history, physical findings, and imaging findings are sufficient for an accurate diagnosis and to omit pathological confirmation; these cases underwent surgical resection or received watchful waiting without biopsy. For example, a simple bone cyst, fibrous dysplasia, or digital bone enchondroma often follow such a course. However, when the lesion does not perfectly fit into any diagnostic category, when the initial image diagnosis appears unconvincing, or when the course of the tumor does not match the initial image diagnosis, biopsy and pathology are indicated, recalling Jaffe’s triangle.

In our case, plain radiography demonstrated a comparatively well-outlined osteolytic lesion, and PET-CT showed no abnormal FDG uptake. Although the lesion was heterogeneously enhanced using Gd.-DTPA on T1-WI MRI, the findings did not lead us to suspect a malignant tumor. Radiologists supposed that the lesion was possibly an enchondroma based on the imaging findings. Nevertheless, the physical and imaging findings did not precisely suggest enchondroma and something was out of place in the diagnosis. Actually, the enhancement pattern on MRI did not match that of enchondroma. Therefore, an incisional biopsy was planned and the results led to the diagnosis of the lesion as synovial sarcoma. Thus, the patient successfully completed the treatment for intraosseous synovial sarcoma, following a careful plan based on the preoperative diagnosis. If curettage was performed based on the initial plan, treatment for this patient would be complicated.

We encounter rare cases in daily practice where malignant tumors mimic benign tumors. In cases where the lesion seems to be benign at first impression, a differential diagnosis may identify findings that do not perfectly fit the preliminary diagnosis of benign tumors. In such situations comprehensive consideration of clinical, radiological, and pathological findings is critically important. Especially, biopsy and pathological findings are important in the case that clinical and radiological findings do not fit into any diagnostic category. These diagnostic processes are crucial for establishing certain diagnosis and providing patients with the best possible treatment.

## Abbreviations

FDG: Fluorodeoxyglucose
FISH: Fluorescence in situ hybridization
Gd.-DTPA: Gadolinium-diethylenetriaminepentaacetic acid
MRI: Magnetic resonance imaging
PET-CT: Positron emission tomography-computed tomography
RT-PCR: Reverse transcription-polymerase chain reaction
T1-WI: T1-weighted images
T2-WI: T2-weighted images
